# The Molecular Adaptive Responses of Skeletal Muscle to High-Intensity Exercise/Training and Hypoxia

**DOI:** 10.3390/antiox9080656

**Published:** 2020-07-24

**Authors:** Jia Li, Yanchun Li, Muhammed M. Atakan, Jujiao Kuang, Yang Hu, David J. Bishop, Xu Yan

**Affiliations:** 1 College of Physical Education, Southwest University, Chongqing 400715, China; jia.li27@live.vu.edu.au; 2 Institute for Health and Sport (iHeS), Victoria University, P.O. Box 14428, Melbourne 8001, Australia; muhammed.atakan@hacettepe.edu.tr (M.M.A.); jujiao.kuang@vu.edu.au (J.K.); david.bishop@vu.edu.au (D.J.B.); 3 China Institute of Sport and Health Science, Beijing Sport University, Beijing 100192, China; lych1216@163.com (Y.L.); huyang@bsu.edu.cn (Y.H.); 4 Division of Nutrition and Metabolism in Exercise, Faculty of Sport Sciences, Hacettepe University, 06800 Ankara, Turkey; 5 Sarcopenia Research Program, Australia Institute for Musculoskeletal Sciences (AIMSS), Melbourne 3021, Australia

**Keywords:** high-intensity exercise, training, hypoxia, skeletal muscle, inflammatory signalling

## Abstract

High-intensity exercise/training, especially interval exercise/training, has gained popularity in recent years. Hypoxic training was introduced to elite athletes half a century ago and has recently been adopted by the general public. In the current review, we have summarised the molecular adaptive responses of skeletal muscle to high-intensity exercise/training, focusing on mitochondrial biogenesis, angiogenesis, and muscle fibre composition. The literature suggests that (peroxisome proliferator-activated receptor gamma coactivator 1-alpha) PGC-1α, vascular endothelial growth factor (VEGF), and hypoxia-inducible factor 1-alpha (HIF1-α) might be the main mediators of skeletal muscle adaptations to high-intensity exercises in hypoxia. Exercise is known to be anti-inflammatory, while the effects of hypoxia on inflammatory signalling are more complex. The anti-inflammatory effects of a single session of exercise might result from the release of anti-inflammatory myokines and other cytokines, as well as the downregulation of Toll-like receptor signalling, while training-induced anti-inflammatory effects may be due to reductions in abdominal and visceral fat (which are main sources of pro-inflammatory cytokines). Hypoxia can lead to inflammation, and inflammation can result in tissue hypoxia. However, the hypoxic factor HIF1-α is essential for preventing excessive inflammation. Disease-induced hypoxia is related to an upregulation of inflammatory signalling, but the effects of exercise-induced hypoxia on inflammation are less conclusive. The effects of high-intensity exercise under hypoxia on skeletal muscle molecular adaptations and inflammatory signalling have not been fully explored and are worth investigating in future studies. Understanding these effects will lead to a more comprehensive scientific basis for maximising the benefits of high-intensity exercise.

## 1. Introduction

Both high-intensity exercise/training and hypoxic training have gained popularity in recent years due to their ability to induce cardiorespiratory and metabolic benefits [1,2], and both have been widely used to improve the performance of athletes [3,4]. Some excellent reviews have summarised the molecular responses to the stress imposed by either high-intensity exercise [5,6] or exposure to hypoxia [7,8,9]. However, little is known about potential synergistic and/or antagonistic effects when these methods are combined. In this review, we explore the underlying mechanisms by which hypoxia, high-intensity exercise, and the combination of the two result in skeletal muscle adaptations (e.g., mitochondria biogenesis, angiogenesis, and inflammatory signalling) 

## 2. High-Intensity Exercise and Physiological Adaptations

### 2.1. Exercise and Training

Exercise is often prescribed to improve both health and performance and to prevent and treat many detrimental health conditions [10,11]. The terms exercise and training are often used interchangeably. To better differentiate these terms in this review, the term “exercise” refers to a single session of physical activity, while the term “training” refers to repeated exercise sessions performed periodically (e.g., for weeks or months).

### 2.2. High-Intensity Exercise and High-Intensity Training

High-intensity exercise is an increasingly popular type of exercise, which is more intense than both moderate- and low-intensity exercise; however, a consensus on its definition has not yet been reached [5]. Different terms have been used previously, including “high-intensity”, “vigorous” [6], “all out” [12,13], “near exhaustion” [1], or “supramaximal efforts” [4,13]. In the current review, we have adopted the definition of high-intensity from our previous work, in which it was defined as an exercise intensity above 75% of the peak power output (PPO) achieved in a graded exercise test (GXT) [5]. Similar to our previous review, high-intensity exercise includes both high-intensity interval exercise (HIIE) and sprint interval exercise (SIE) [5]. High-intensity interval exercise has been defined as short bursts of vigorous exercise, with periodic intervals of rest or low-intensity exercise [14]. A meta-analysis from 2016, which included articles containing “high-intensity exercise”, reported exercise intensities varying from 80% to 250% of maximal oxygen uptake ($\overset{\cdot }{V}$O_2max_), and durations of 20–240 s [15]. We specify HIIE as “near maximal” efforts (~75–95% PPO) lasting 1–5 min, separated by resting periods, while SIE has a duration of no more than 60 s and exercise intensities exceeding the PPO recorded from a GXT (i.e., “supramaximal”). The terms high-intensity interval training (HIIT) and sprint interval training (SIT) refer to the repetition of HIIE and SIE exercise sessions, respectively.

Compared to continuous endurance exercise, short exercise durations are suggested to be more convenient for individuals who lack time and motivation [16]. Furthermore, when energy expenditure is matched, HIIT has been shown to benefit individuals with cardiovascular conditions more than moderate-intensity continuous training (MICT) [17]. Although the exercise intensity is very high, the low intensity during rest intervals will make the body prone to adapt HIIT more than continuous training [18]. 

### 2.3. Commonly Used High-Intensity Exercise/Training Prescriptions

Growing evidence from a range of different protocols available suggests that high-intensity exercise is a time-efficient exercise strategy to improve cardiorespiratory and metabolic health [19,20]. Of the SIE protocols utilised, the most common is the Wingate protocol, consisting of 30 s “all-out” cycling efforts against a supra-maximal workload and which lasts ∼20 min, with a total of 2 to 3 min of intense exercise in total. However, the Wingate protocol is extremely demanding and may not be safe to be prescribed to all populations [14]. Therefore, new SIT protocols were designed with a wider application to individuals who are overweight/obese, older, sedentary, or at higher risk for cardiometabolic disorders, and patients with coronary artery disease and type 2 diabetes (T2D). Recent research has focused on the potential for other models of SIT, which may be more feasible whilst remaining time-efficient [21]. For example, performing 10 × 60 s at $\overset{\cdot }{V}$O_2max_, interspersed by 75 s of rest, has been shown to increase the activity of mitochondrial enzymes and also exercise capacity [21,22], improve substrate metabolism [23], and reduce hyperglycemia [21] as well as the feelings of nausea and discomfort usually reported after the Wingate protocol. Another high-intensity exercise protocol commonly used in the literature involves 4 × 4 min at 85–90% of $\overset{\cdot }{V}$O_2max_, separated by 2 min of rest; this was shown to provide a sufficient stimulus to improve exercise capacity, as well as the whole-body and skeletal muscle capacity for fatty acid oxidation [24].

## 3. Skeletal Muscle Molecular Responses to High-Intensity Exercise/Training 

### 3.1. High-Intensity Exercise/Training and Skeletal Muscle Mitochondrial Biogenesis

Mitochondria are considered the powerhouse of the cells. Mitochondrial biogenesis has been defined as “the making of new components of the mitochondrial reticulum” [5,25]. Mitochondria can increase the content of their components or fuse with other mitochondria [26], depending on the needs of the cell. There is a decrease in the number and function of muscle mitochondria during aging, while exercise has been shown to alleviate this reduction [27].

From the onset of exercise, several signalling molecules are induced, including proliferator-activated receptor gamma coactivator 1 alpha (PGC-1α). PGC-1α is considered to be a master regulator of mitochondrial biogenesis and is able to effectively coordinate mitochondrial biogenesis through binding to and activating transcription factors, such as mitochondrial transcription factor A (TFAM) [28] and nuclear respiratory factors (NRFs) [29]. 

Previous studies have shown that PGC-1α protein increased in human muscle during a prolonged exercise session [30] and remained upregulated 24 h after the exercise session [31]. Comparatively, when matched for workload and intensity, intermittent exercise (30 × 1-min intervals at 70% $\overset{\cdot }{V}$O_2peak_, separated by 1-min of recovery) induced a greater activation of PGC-1α signalling pathways compared to a single bout of continuous exercise [30]. Our previous data in human skeletal muscle also demonstrated that a single session of HIIE (8 × 2-min intervals at ~85% of PPO) increased the PGC-1α mRNA expression [32] and the content of proteins related to mitochondrial biogenesis [33]. This has also been demonstrated elsewhere with a different exercise protocol; in human skeletal muscle, the mRNA and nuclear content of PGC-1α increased 3 h after a single session of HIIE (5 × 4-min intervals at ~80% of PPO), while HIIT twice-daily for 20 consecutive days blunted this response to HIIE [34]. Others have reported an increase in the nuclear content of PGC-1α protein immediately after a single session of SIE (4 × 30 s), but not after a session of continuous exercise at 63% of the PPO [35]. However, another study reported that a session of SIE (4 × 30 s Wingate intervals, interspersed with 4 min of rest) resulted in similar elevations in PGC-1α mRNA when compared to a session of continuous all-out exercise [36]. 

During long-term training, AMP activated protein kinase (AMPK, a heterotrimeric protein complex and a mediator of mitochondrial biogenesis) and other signals facilitate skeletal muscle adaptations, including an upregulation of PGC-1α [37], to accomplish a metabolic transition to oxidative metabolism. An increase in PGC-1a is able to induce a variety of target genes, one of them being citrate synthase (CS) [38]. Eight weeks of continuous (30 min of continuous running at 60% of the maximal work rate) or intermittent endurance exercise (3 × 10-min intervals at 60% of the maximal work rate with 2 h between intervals) performed five times per week resulted in similar increases in CS protein in rodents [39]. Previous results from our group support that training intensity is critical for improving mitochondrial function with training, while training duration is essential for improving the mitochondrial content [40]. While four weeks of HIIT at 80–95% of the PPO did not increase the skeletal muscle mitochondrial respiratory function, four weeks of SIT led to a higher mitochondrial function (i.e., maximal mitochondrial respiration) [41]. A separate study also showed similar findings in mitochondrial respiratory function; however, a modest but significant increase in CS activity was observed after four weeks of HIIT, suggesting an increase in mitochondrial content [42]. The failure to detect significant changes in mitochondrial function could be due to technical variations [43] or genetic factors [32,33,44,45,46,47]. 

### 3.2. High-Intensity Exercise/Training and Muscle Fibre Type Composition

The muscle fibre type composition affects oxygen consumption during exercise. Type I (slow-twitch fibres) and type IIa muscle fibres (fast-twitch oxidative fibres) have a high density of mitochondria and a high oxidising power, while type IIx and IIb (fast-twitch glycolytic) fibres have a lower mitochondrial density [48]. Limited research has specifically focused on the effects of HIIT on different muscle types. We have previously suggested that high-intensity exercise induces mitochondrial biogenesis in type II fibres, while the mitochondrial content of type I fibres is more related to low-intensity continuous exercise [40]. However, a study involving sedentary young women who performed six weeks of HIIT (~90% HR_max_) revealed that short term HIIT was able to increase the mitochondrial content of both type I and type II fibres in the vastus lateralis muscle [49].

PGC-1α is known to contribute to skeletal muscle fibre type transformation in rodents by reducing the ratio of glycolytic (type IIb) to oxidative fibres (type I and type IIa). It has been shown that over-expressing PGC-1α in mouse plantaris muscle increases the mitochondrial content and respiratory function, with a transition rate of 20% for type IIa and 10% for type I fibres from the fast-twitch type IIb muscle fibres, respectively [50,51]. It was further reported that mice lacking PGC-1α had fewer mitochondria, which was associated with a decreased endurance exercise capacity and fatigue-resistant type I muscle fibres [51]. Since PGC-1α can be induced by both HIIE and HIIT [29], the above study provided indirect evidence that high-intensity exercise could affect muscle fibre types. 

### 3.3. High-Intensity Exercise/Training and Skeletal Muscle Angiogenesis

Angiogenesis refers to the growth of new blood vessels from original vessels during natural development, reproduction, or tissue repair [52]. Angiogenesis is linked to exercise, aging, and cancer [53,54]. Angiogenic factors are regulated by vascular endothelial growth factor (VEGF), fibroblast growth factor (FGF), and hypoxia-inducible factors (HIFs). When these angiogenic factors bind to their receptors on endothelial cells, signals within these cells are initiated to promote the growth and survival of new blood vessels [55]. Two common downstream targets are angiopoietin-1 (ANGPT-1) and angiopoietin-2 (ANGPT-2). ANGPT-1 is known as a pro-angiogenic factor [56]. While ANGPT-2 has been reported as an antagonist of ANGPT-1, it is still important for angiogenesis [57]. Excessive angiogenesis has been linked with some malignant diseases, such as cancer, diabetic retinopathy, and preeclampsia. Interestingly, VEGF-induced angiogenesis has been linked with an increase in mitochondrial respiratory function [58]. Moreover, PGC-1α has been shown to regulate the expression of myoglobin, an oxygen storage hemoprotein and facilitator of intracellular oxygen transport [59].

Exercise induces a range of adaptations, including an upregulation of angiogenesis that, in turn contributes to exercise adaptations [54]. Several studies have evaluated changes associated with angiogenesis after exercise. After eight weeks of moderate-intensity incremental treadmill exercise, the VEGF mRNA expression increased in rat skeletal muscle [60]. Others have shown blood VEGF and ANGP-1 to be significantly increased after eight weeks of resistance training, and suggested that moderate-intensity resistance training might lead to higher angiogenesis compared to high-intensity resistance training [61]. The interstitial content of VEGF protein has been shown to increase after a session of moderate-intensity exercise (60 min of exercise at ~64% of $\overset{\cdot }{V}$O_2max_) and a session of SIE (24 × 1 min, at 117% of $\overset{\cdot }{V}$O_2max_, separated by 1.5 min of passive rest); however, the increase with moderate-intensity exercise was greater than that of SIE [62]. In human skeletal muscle, endurance exercise has been shown to decrease the protein content of anti-angiogenic regulators, together with an increased capillarity in the muscle [39].

High-intensity exercise has been shown to induce angiogenesis [63]. A previous study compared the effects of four weeks of HIIT (one-legged extensor exercise three times per week, with 1 min at 90% of $\overset{\cdot }{V}$O_2max_ and 30 s rest for a total of 1 h) and SIT (one-legged extensor exercise, with 1 min at 150% of $\overset{\cdot }{V}$O_2max_ and 3 min of rest for a total of 1 h, three to four times per week) on angiogenesis, and reported similar increases in the density of capillaries and the presence of proliferating endothelial cells [63]. An acute session of one-legged knee-extensor exercise at ~70% of the maximal load has also been shown to induce VEGF mRNA expression in human skeletal muscle [64], while four weeks of training (performed five times per week) attenuated the acute exercise-induced upregulation of VEGF mRNA [64]. 

### 3.4. Summary

High-intensity exercise leads to a number of skeletal muscle adaptations, such as an upregulation of mitochondrial biogenesis and angiogenesis, as well as muscle fibre type transformation. PGC-1α and VEGF are two of the key mediators of these adaptations. 

## 4. Physiological Adaptations to Acute and Long-Term Hypoxia

### 4.1. Acute Hypoxia and Physiological Adaptations

“Physiological hypoxia” is defined as a condition in which the oxygen concentration in the human body is significantly lower than that in the atmosphere [65]. It can be caused by some physiological responses, such as vasodilation and increasing blood flow [66]. Hypoxia has long been known to affect exercise performance. Both the PPO and $\overset{\cdot }{V}$O_2peak_ obtained from GXTs were lower when the inspired oxygen fraction (F_i_O_2_) was reduced to 14% (equivalent to a 3200 m altitude) [67]. Furthermore, the peak heart rate and $\overset{\cdot }{V}$O_2max_ achieved from GXTs were also lower with acute hypoxia (F_i_O_2_ of 10.4%, equivalent to a 5500 m altitude) [68]. The lactate threshold (LT), as well as the PPO and $\overset{\cdot }{V}$O_2max_, were also reduced during an incremental exercise test with an F_i_O_2_ of 12% (equivalent to a 4450 m altitude) [69]. 

### 4.2. Long-Term Hypoxia and Physiological Adaptations

In the 100-m race at the 1968 Olympics in Mexico City (elevation 2250 m), male and female athletes who were living in a high altitude were reported to have a time advantage of about 0.19 s and 0.21 s, respectively, compared to athletes who were living at sea level [70]. Consequently, these observations triggered attention towards research into the long-term effects of hypoxia on exercise performance. Interestingly, it has been shown that high-altitude natives (also known as “highlanders”) and sea-level natives have a similar $\overset{\cdot }{V}$O_2max_ in normoxic conditions (where the FiO_2_ is 20.9%), but that highlanders can attain a greater $\overset{\cdot }{V}$O_2max_ when the oxygen availability is reduced at altitude exposures of 3600 m (FiO_2_ of 13.3%) [71]. High-altitude natives usually have an enhanced performance and/or increased physical work capacity [72,73]. Highlanders from Tibet (3700–4000 m) seem to possess a better economy of walking, cycling, and treadmill running compared to acclimatised lowlanders (sea level) [74]. These advantages of the high-altitude natives may have occurred as a result of both a genetic and a developmental basis [72].

Sea-level natives who become acclimatised to high altitudes have been shown to improve their physical work capacity and exercise performance, although not to the extent of highlanders [71]. Altitude training is now mostly used with endurance training to increase the exercise capacity at sea level and to improve adaptations during competitions in high-altitude areas [75]. Training at a high altitude has been shown to improve the endurance performance of sea-level natives in hypoxic conditions; however, the improvements in their endurance performance was less conclusive once back at sea-level [76].

## 5. Skeletal Muscle Molecular Response to Hypoxia 

Under conditions of insufficient oxygen supply in the human body, oxygen-sensing mechanisms are activated to restore oxygenation and adapt quickly to the hypoxic conditions by initiating survival responses such as increased respiration and blood flow [77]. The major oxygen sensing mechanism is the Hypoxia Inducible Factor (HIF), which is dependent on prolyl hydroxylases (PHDs). HIF-1, which was first reported in 1997, is a transcription factor of the human gene encoding erythropoietin (EPO) and is the main regulator of genes responsive to hypoxia [78]. For the first time, Semenza et al., predicted the existence of a hypoxia-dependent transcription factor [79] and identified HIF-1 as a transcription factor for the cellular hypoxia response [80]. HIF-1 is composed of two subunits: an oxygen-regulated subunit (HIF-1α) and a constitutively expressed subunit (HIF-1β). 

### 5.1. Hypoxia with and without Exercise/Training, and Mitochondrial Biogenesis

The reduction in oxygen availability under hypoxia requires cells to change their metabolism to adapt to the catabolic and anabolic reactions that rely on the availability of ATP normally supplied by mitochondrial Oxidative Phosphorylation (OXPHOS) [81]. HIF-1α signalling reduces cell dependence on oxygenated energy products by downregulating OXPHOS [82]. Activated HIF-1α has an important function in metabolic transition under hypoxia [83]. HIF-1α target genes are involved in oxygen transport, glycolysis, glucose transport, and satiety [84]. A study reported that after a 43-day Himalayan Expedition (with 23 days above 5000 m), the slow isoform I of both heavy and light myosin subunits increased and the fast isoform IIa decreased, suggesting chronic hypoxia results in a fast-to-slow muscle fibre transition, which could lead to a faster activation of mitochondrial oxidative metabolism [85].

Both hypoxia and exercise are able to increase HIF-1α accumulation [78]. A recent study showed that the skeletal muscle HIF-1α protein content was 120% higher after hypoxia exposure, and was further induced by exercise [86]. Compared to resting in normoxia, exercise in hypoxia raised the HIF-1α protein expression approximately 2.5-fold [86]. When the oxygen supply is insufficient, HIF-1α target genes improve oxygen transport by EPO-mediated erythropoiesis and VEGF-induced angiogenesis mechanisms, and mediate skeletal muscle adaptions to endurance training through optimised glucose transport and glycolytic enzyme activity. Finally, training in hypoxia increases PGC-1α mRNA expression [87], which induces mitochondrial biogenesis. In addition, it has been suggested that the combination of hypoxia and exercise may have a synergistic effect on body composition and metabolism [2]. 

### 5.2. Hypoxia and Angiogenesis

Angiogenesis is a hallmark adaptation to hypoxia in cells and tissues [88]. Most transcriptional responses to hypoxia are mediated by hypoxia-inducible transcription factors [88], including HIF-1α and HIF-2α. HIFs have been shown to upregulate the pro-angiogenic factor ANGPT-1 and to downregulate ANGPT-2 [89]. A previous study reported that HIF-1α regulated VEGF in response to hypoxia more than HIF-2α [90]. The current literature suggests that hypoxia and HIF-1 expression in adult organisms may promote angiogenesis in the following ways: (1) activating angiogenic genes and their receptors such as VEGF [91], (2) regulating proangiogenic chemokines and receptors [92], and (3) enhancing endothelial cells and regulating genes in the cell cycle and DNA replication [93]. All of these findings offer evidence for the effects of HIF-1 on angiogenesis [88].

VEGF, which promotes the proliferation of endothelial cells, is also a hypoxia-adaptive gene. The translocation of proliferated endothelial cells into the extracellular matrix has an important effect on angiogenesis. VEGF and EPO respond to acute changes in oxygen demand in human skeletal muscle, suggesting that oxygen-sensitive pathways could be relevant for adaptation to physical activity by increasing capillary growth [94]. In fact, besides regulating mitochondrial biogenesis, PGC-1α protein also increases the VEGF mRNA expression and subsequent angiogenesis [95]. On the other hand, the transcription factors HIF-1α and HIF-2α also regulate the expression of VEGF in response to hypoxia [96]. Most of the evidence on hypoxia and angiogenesis is from studies in cancer and angiogenesis. These studies found that acute hypoxia may result in a dysregulation of tumour vascular systems. In chronic hypoxia, HIF-1α regulates the proangiogenic activities of VEGF, which control the expression of a multitude of genes, inducing angiogenesis [97]. This can ultimately trigger the adaptive mechanisms of angiogenesis to optimise oxygen delivery [98]. VEGF has been reported in many studies to be the most remarkable factor that stimulates angiogenesis [99]. Of note, hypoxia is known to induce the expression of myoglobin in skeletal muscle [100], an alternate way to increase oxygen availability.

### 5.3. High-Intensity Exercise/Training in Hypoxia and Angiogenesis

It has been shown that the combination of hypoxia and exercise training are capable of improving muscle oxygen delivery and metabolism [101]. Data from an animal study have suggested that exercise-induced angiogenesis can alleviate obesity-induced tissue hypoxia [102]. In human skeletal muscle, 45 min of one-legged knee-extension exercise (~26% of one-legged peak load) increased the VEGF mRNA, which was further increased in hypoxia (induced via restricted blood flow by ~15–20%) [103]. Six weeks of high-intensity training twice per week (involving two bouts of 12–20 min of running at ~92% of the maximal running speed, separated by 5 min of recovery) in normobaric hypoxia (F_i_O_2_ of 14.5%, equivalent to 3000 m) increased the mRNA of HIF-1α, PGC-1α, and CS in endurance athletes [87]. Similar findings were also shown after six weeks of moderate-intensity (65.6% of the PPO) and low-intensity (52.4% of the PPO) endurance training, performed five times per week in normobaric hypoxia (F_i_O_2_ of 12.9%, 3500 m); both training programs increased the HIF-1α mRNA expression and mitochondrial density [2]. The capillary density also increased after six weeks of moderate-intensity exercise [2].

In horse skeletal muscle, four weeks of high-intensity training (2 min of running at 100% $\overset{\cdot }{V}$O_2max_, three times per week) in hypoxia (F_i_O_2_ of 15%, ~2620 m) resulted in a greater increase in HIF-1*α* and VEGF mRNA expression compared to training in normoxia, and was associated with a higher capillary density [104]. In a human study, eight weeks of resistance training (twice per week, five sets of 10 repetitions at 70% of pretraining one-repetition maximum, separated by 90 s of rest between sets) was performed in normoxic and hypoxic conditions (F_i_O_2_ of 14.4%, equivalent to 3000 m) [105], the plasma VEGF protein and capillary-to-fibre-ratio increased only after hypoxic resistance training [105]. A recent study investigated the effect of HIIT on the serum concentrations of pro-angiogenic factors in hypoxia, and found that HIIT alone resulted in a significant increase in exercise-induced elevations in serum VEGF, but exercise in hypoxia did not further influence the VEGF levels [106]. 

### 5.4. Summary

Hypoxia leads to several skeletal muscle adaptations, including the modification of mitochondrial biogenesis and angiogenesis; PGC-1α, HIF-1α, and VEGF are involved in these adaptations. Furthermore, high-intensity exercise in hypoxia can further induce HIF-1α and PGC-1α, while its effects on VEGF need to be further explored.

## 6. Inflammatory Response to Exercise/Training and Hypoxia

Inflammation is a complicated physiological process associated with the activation of several signalling pathways, usually in response to stress [107]. Chronic low-grade inflammation is associated with metabolic disorders, such as obesity, insulin resistance, and T2D [108,109,110,111]. This inflammatory state is characterised by increased levels of circulating inflammatory markers, such as tumour necrosis factor (TNF), interleukin-6 (IL-6), and C-reactive protein (CRP) [112]. Of note, the inflammatory response consists of both anti- and pro-inflammatory mediators [107]. TNF-α and interleukin-1β (IL-1β) are examples of pro-inflammatory cytokines, while IL-10 and interleukin-1 receptor antagonist (IL-1ra) are anti-inflammatory cytokines [113]. Despite high levels of IL-6 having been associated with obesity and T2D, IL-6 also exhibits anti-inflammatory effects during exercise [113,114]. Toll-like receptors (TLRs) are highly conserved transmembrane proteins with important functions in detecting exogenous microbial pathogens and endogenous molecules, which are released after tissue damage [115]. TLR signalling has a critical role in mediating systemic inflammation, and its activation leads to the elevated expression and release of pro-inflammatory cytokines [116].

Mitochondria have long been linked with inflammation [117,118,119,120,121,122,123]. The endosymbiotic theory of mitochondrial origin supports the role of mitochondria in the activation of the immune system, and therefore inflammation and the pathogenesis of inflammatory diseases [117]. The immune signalling receptor TLR has been associated with mitochondrial functions, suggesting the role of mitochondria in the initiation and development of inflammation [117]. Mitochondria-associated membranes are linked with inflammation-mediated diseases [118]. The mitochondrial outer membrane permeabilisation has recently been shown to have pro-inflammatory effects via pro-inflammatory NF-κB signalling [121]. Through regulating the energetic state of immunological synapses between dendritic cells and lymphocytes, mitochondria can direct the inflammatory response toward immunotolerance or immunogenicity [119]. Mitophagy, the selective degradation of dysfunctional mitochondria by autophagy, can dampen inflammation and prevent unnecessary cell loss [120].

Inflammation seems to be linked with muscle fibre composition. The pro-inflammatory cytokine profile is different between the soleus (oxidative) and extensor digitorum longus (EDL) (glycolytic) muscles of mice [124]. Soleus muscle regeneration is associated with elevated and prolonged inflammation as compared to EDL [124]. In mouse skeletal muscle, an inhibition of the slow-to-fast muscle fibre type transition by pyrroloquinoline quinone was reported to be due to a decrease in the expression of cytokine genes [125]. After treadmill running, mice with muscle-specific PGC-1α knock-out showed a shift from oxidative type I and IIa toward type IIx and IIb muscle fibres, and this was associated with elevated markers of inflammation [51]. TLR4 signalling is essential for lauric acid-induced glycolytic muscle fiber formation [126]. A downhill running-based overtraining protocol resulted in changes in the inflammatory markers and muscle fibre composition in mice [127]. Chronic inflammation is able to increase the abundance of type II muscle fibres in the diaphragm of mice [128].

Angiogenesis has also been linked with inflammation, which can lead to further angiogenesis [129], and vice versa [130]. Abnormal ischaemia-induced angiogenesis is associated with a transiently increased angiogenesis in the ischaemic skeletal muscle of mice [131]. The angiopoietin-like protein (ANGPTL) protein family is involved in both angiogenesis and inflammation [132]. Obesity-associated inflammation has been reported to promote angiogenesis (and breast cancer) via ANGPTL-4 [133]. Inflammatory reactions are able to regulate angiogenesis through the interplay between HIF1, HIF2, NF-κB, and nitric oxide [129].

### 6.1. High-Intensity Exercise and Inflammation 

Exercise has long been recognised to have anti-inflammatory effects [112,113,114]. There are at least three possible mechanisms proposed, including a lower expression of TLRs on macrophages and monocytes, a higher production and release of anti-inflammatory cytokines from contracting muscle, and a reduction in the visceral fat mass [112]. Secretory peptides from skeletal muscle have been termed myokines [134], many of which are induced by exercise, such as interleukin 6 (IL-6), IL-15, and brain-derived neurotrophic factor (BDNF) [135]. Interestingly, some myokines show anti-inflammatory effects after a single session of exercise [113].

#### 6.1.1. High-Intensity Exercise and Inflammation

In healthy humans, cycling at 75% of the $\overset{\cdot }{V}$O_2max_ for 1.5 h reduced the monocyte TLR4 by 32% immediately after exercise and by 45% 1 h after exercise [136]. In both healthy and diabetic individuals, a single session of HIIE (7 × 1 min at 85% PPO, interspaced with 1 min of recovery) reduced TLR2 in monocytes immediately after and 1 h after exercise [137].

Of all the myokines, IL-6 was identified as the first responder to acute exercise and was five-fold higher after only 30 min of running at 75% of the $\overset{\cdot }{V}$O_2max_ [138]. Moreover, IL-6 can increase 100-fold after a marathon [139]. IL-6 levels typically peak at the end of or shortly after exercise, followed by a rapid decrease toward the baseline [114]. The response of IL-6 to exercise depends on the intensity [140] and duration of exercise [114,141]. As previously mentioned, IL-6 was regarded as a pro-inflammatory cytokine, and was thought to be an indicator of muscle damage after exercise [114]. However, it was soon realised that IL-6 is involved in a different inflammatory cascade in response to exercise than sepsis (a life-threatening condition caused by a dysregulated host response to infection [142]). During sepsis, there is a marked increase in typical pro-inflammatory cytokines, such as TNF-α and IL-1, which subsequently induce the production of IL-6 [113]. However, during exercise there is a lack of increase in TNF-α and IL-1, and instead IL-6 stimulates the production of IL-1ra and IL-10, two common anti-inflammatory cytokines, from blood mononuclear cells [143]. IL-1ra inhibits the pro-inflammatory effects of IL-1β [144], and IL-10 acts to downregulate adaptive immune responses, aiming to limit and ultimately terminate inflammatory responses [145]. Direct evidence also arises from a study in humans, in which IL-6 infusion suppressed endotoxin-induced TNF-α elevation, with similar effects observed after 3 h of ergometer cycling [146].

In addition to IL-6, epinephrine may also mediate the anti-inflammatory effect of exercise [114]. A single session of exercise increased the levels of epinephrine [114], and epinephrine infusion has been shown to suppress endotoxin-induced TNF-α elevations in humans [147]. Exercise induces an increase in cortisol, which has commonly been used in the treatment of inflammatory disorders, providing further support for the anti-inflammatory effects of exercise [114]. Interestingly, exercise-induced elevations in the cortisol level are IL-6 dependent, but independent of epinephrine [143]. Anti-inflammatory cytokines IL-1ra, IL6, and IL-10 increased significantly 30 min after an ironman triathlon race, without changes in the pro-inflammatory cytokine IL-1β [148]. 

Early studies suggested that exercise might lead to inflammation in skeletal muscle. Animal studies reported that the TNF-α, IL-1β, and IL6 mRNA levels were elevated in mouse skeletal muscle 24 h after an exhaustive exercise, while the depletion of microphages [149] and neutrophil [150] blunted the elevation. One study reported that a session of eccentric exercise resulted in microphage infiltration into human skeletal muscle, similar to what occurs in inflammatory muscle disease [151]. Inflammatory cell infiltration was also observed 10 days after 45 min of eccentric cycling at a high intensity (15 min at 90%, 80%, and 70% of the PPO, with 5 min of rest in between) [152]. Others have shown that 45 min of downhill running (16% incline, 70% of the maximum heart rate) led to an increase in the muscle IL-1β immediately after exercise, which remained elevated five days after exercise [153]. However, these findings have since been challenged [154,155]. When compared with the non-exercise control group, the expression of IL-1β in skeletal muscle increased similarly after 30 min of eccentric cycling at or near a maximal work rate (250 to 300 W) [155]. The eccentric cycling performed by the exercise group, and the process of muscle biopsies, did not result in T cell infiltration into the human skeletal muscle [155]. It has been argued that the discrepancies are due to the lack of non-exercising control groups in the previous studies, while the observed muscle inflammation could be due to intramuscular injections or the muscle biopsy procedures [154]. 

#### 6.1.2. High-Intensity Training and Inflammation

Training has been shown to decrease TLRs. In both young and old inactive participants, the TLR4 expression in monocytes was reduced after twelve weeks of combined endurance and resistance training, performed three days/week [156]. Six weeks of eccentric training resulted in a lower TLR4 protein level in response to an acute eccentric bout [157]. In diet-induced obese mice, 16 weeks of endurance training (60 min/day, 5 days/week) led to lower TNF-α, IL-6, and TLR4 mRNA expressions in adipose tissue [158], as well as less microphage [159,160] and neutrophil infiltration [161] into the adipose tissue, indicating less adipose tissue inflammation. Additionally, in elderly women, 12 weeks of resistance training were associated with lower circulating levels of TNF-α [162]. Notably, endurance training and resistance training exert different effects on genes related to inflammation, based on a transcriptional profile analysis in human skeletal muscle [163].

The anti-inflammatory effects of training could be related to the decreased accumulation of abdominal and visceral fat, which are the main sources of pro-inflammatory cytokines [112]. A reduction in daily physical activity from 10,000 to 1500 steps for as little as 14 days has been shown to significantly increase the intra-abdominal and visceral fat mass. The accumulation of both abdominal and visceral fat is accompanied with low-grade chronic inflammation [114]. Training-induced reductions in fat mass can be seen in both males and females, regardless of age, even without changes in body mass [14], and have been associated with lower pro-inflammatory cytokines in the blood [114]. 

The anti-inflammatory effects of training might also be mediated, at least partially, by IL-15 [114]. In human muscle, the IL-15 expression was higher after training [164], while in mice the overexpression of IL-15 prevented the accumulation of visceral fat [165]. Additionally, in mice IL-15 administration led to a 36% decrease in circulating leptin [166], a hormone that is secreted from adipocytes and closely linked to inflammation [167]. Furthermore, the skeletal muscle-specific overexpression of IL-15 has been shown to reduce circulating leptin in mice [168]. It has been suggested that IL-15 induction (for example, by training) may decrease or even inhibit the negative effects of TNF-α in obesity or T2D, which are both associated with low-grade chronic inflammation [169]. It seems that training intensity might affect the inflammatory response; six weeks of MICT (40% of PPO, 3 days/week) was shown to decrease the pro-inflammatory cytokine, TNF-α, whereas this reduction was not observed after six weeks of HIIT (10 × 1-min at 80% of the PPO, 3 days/week) [170]. Interestingly, the anti-inflammatory cytokine IL-6 was higher after six weeks of HIIT but not MCT [170].

In mouse muscle, two months of continuous running (80% of the $\overset{\cdot }{V}$O_2max_, 30 min/day, 5 days/week) was shown to rescue the upregulations of TNF and IL-1 mRNA induced by cigarette smoke exposure [171]. Twelve weeks of resistance training decreased the transcription of genes involved in monocyte recruitment, but upregulated the transcription of genes involved in the switch from a pro- to an anti-inflammatory macrophage phenotype, following a single session of resistance exercise [172]. Notably, although once thought to be detrimental to muscle regeneration, the initial pro-inflammatory signalling response to muscle injury is a critical part of the recovery process involving satellite cell activation and muscle regeneration [173].

### 6.2. Hypoxia and Inflammation

Hypoxia and inflammation are tightly interconnected [77,174]. On the one hand, hypoxia can induce inflammation as evidenced by the increased levels of circulating pro-inflammatory cytokines with mountain sickness [175]. A three-night stay at 3400 m above sea level (F_i_O_2_ of 13.6%) has been shown to increase the levels of circulating pro-inflammatory cytokines, such as C-reactive protein and IL-6 [176]. Mice exposed to short-term, extreme hypoxic conditions ((F_i_O_2_ of 8% for 8 h) exhibit mucosal inflammation and elevated circulating pro-inflammatory cytokines [177]. Obesity is usually accompanied by adipose tissue hypoxia, which is associated with chronic low-grade systemic inflammation [178]. On the other hand, tissues with inflammation often become hypoxic [77]. A good example is inflammatory bowel disease, in which the mucosa becomes more hypoxic than in normal conditions [179], which is accompanied by a higher protein content of HIF-1α [180]. Besides the well-accepted concept that hypoxia causes inflammation, hypoxia might also possess some anti-inflammatory effects [174]. A short stay at 3400 m above sea level (F_i_O_2_ of 13.6%) also resulted in an increase in anti-inflammatory cytokine IL-1ra [176]. The stabilisation of HIF-1α, the main mediator of hypoxia signalling, has been demonstrated to control excessive inflammation [181]. Notably, a study found a subtle but non-significant decrease in TNF-α and IL-6 after two hours at simulated hypoxia at 4500 m (F_i_O_2_ of 11.8%) [182]. 

### 6.3. High-Intensity Exercise/Training in Hypoxia and Inflammation

Only a handful of studies have examined the effects of exercise in hypoxia on inflammation. One study compared the effects of exercise at different intensities in normoxia and hypoxia (2800 m, F_i_O_2_ of 14.65%) on pro-inflammatory cytokines, and found no significant differences in the TNF-α or IL-1 after exercise at 40% or 60% of the $\overset{\cdot }{V}$O_2max_ [183]. Another study investigated the level of cytokines after an exercise bout at 70% of the $\overset{\cdot }{V}$O_2max_ performed until exhaustion, and detected no changes in TNF-α immediately after or 2 h after the exercise session, while the IL-6 was higher at both time points [182]. One study compared the effects of a 60 min exercise session at 70% of the $\overset{\cdot }{V}$O_2max_ in normoxia and hypoxia (4200 m, F_i_O_2_ of 12%) on cytokines and muscle damage markers [184]. The TNF-α increased to a similar level immediately after exercise in both normoxia and hypoxia, but remained higher one hour after exercise only in the hypoxic condition [184]. The anti-inflammatory cytokines IL-10 and IR-1ra were both higher in normoxia and hypoxia one hour after exercise, while the IL-6 was higher immediately after and one hour after exercise in the hypoxic condition [184]. Another study reported no differences in IL-6 immediately, 60 min, and 120 min after a combination of HIIE (10 × 3 min running at 95% of the $\overset{\cdot }{V}$O_2max_ with 60 s of active rest at 60% of the $\overset{\cdot }{V}$O_2max_) and 30 min of continuous running (at 85% of the $\overset{\cdot }{V}$O_2max_) under either hypoxic (F_i_O_2_ of 14.5%) or normoxic conditions [185]. Interestingly, the level of myoglobin in the blood, an indicator of muscle damage, was even lower in the hypoxic condition [185]. 

## 7. Conclusions

**Figure 1:**
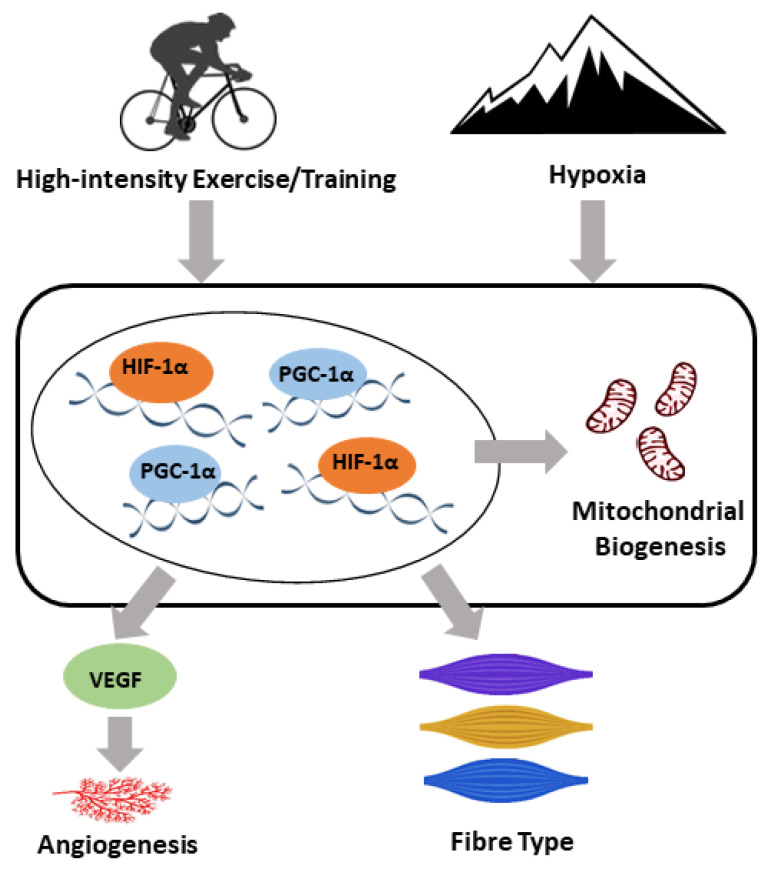
A speculative model for the molecular adaptive responses of skeletal muscle to both high-intensity exercise/training and hypoxia. Both exercise/training and hypoxia induce a range of adaptations, including an upregulation in angiogenesis and mitochondrial biogenesis and a shift in the skeletal muscle fibre type. Peroxisome proliferator activated receptor gamma coactivator 1 alpha (PGC-1α), hypoxia-inducible factor 1-alpha (HIF-1α), and vascular endothelial growth factor (VEGF) play important roles in the regulation of the adaptive response to both high-intensity exercise/training and hypoxia within skeletal muscle.

High-intensity exercise, especially interval exercises, has gained popularity recently. Even though no consensus on the definition of “high-intensity” has been reached, exercise intensities higher than 75% of the VO_2max_ or PPO are commonly used. High-intensity exercise leads to similar, if not greater, improvements in skeletal muscle metabolic adaptations, cardiorespiratory fitness, vascular function, and body composition than moderate-intensity exercises, and is more time efficient. Within skeletal muscle, high-intensity exercise is associated with similar or great adaptations in mitochondrial biogenesis and angiogenesis in a muscle fibre type-specific manner. The main molecular mediators involved in these adaptations include PGC-1α, HIF1-α, and VEGF, which are also linked to the hypoxic response [96]. A speculative model has been proposed for the role of PGC-1α, HIF1-α, and VEGF in the adaptive responses of skeletal muscle to both high-intensity exercise and hypoxia (Figure 1). 

**Figure 2:**
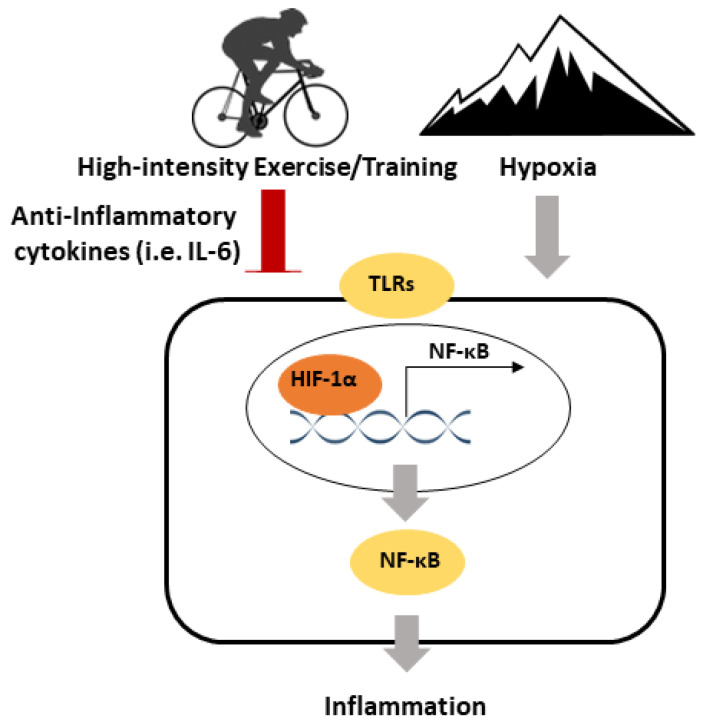
A speculative model for the inflammatory responses to high-intensity exercise/training and hypoxia. Exercise/Training exhibits anti-inflammatory effects via the induction of anti-inflammatory cytokines and downregulating toll-like receptor (TLR) signalling. Hypoxia is pro-inflammatory and mediates the upregulation of TLR signalling. HIF-1α is important in regulating the inflammatory response to high-intensity exercise/training and hypoxia.

Exercise is also known to exhibit anti-inflammatory effects. A single exercise session leads to an immediate increase in IL-6 (an anti-inflammatory myokine), without an increase in the pro-inflammatory cytokines TNF-α and IL-1β. Training has been associated with the reduced activation of TLR signalling and reductions in the abdominal and visceral fat, which can both lead to lower inflammation. Both a single exercise session of exercise and training mediate reductions in inflammatory signalling in skeletal muscle, which may be related to the expression of anti-inflammatory myokines, as well as reductions in TLR signalling. Hypoxia is associated with an upregulation of inflammatory signalling, such as a higher pro-inflammatory cytokines. On the other hand, HIF1-α is critical to control excessive inflammation. A speculative model has been proposed for the inflammatory responses to high-intensity exercise and hypoxia (Figure 2). Few studies have explored the effects of exercise on inflammatory signalling in hypoxia, but this limited research reported no differences in pro-inflammatory cytokines, and increases in anti-inflammatory cytokines. Future studies are required to explore the effects of high-intensity exercise under hypoxia on inflammatory signalling, especially in a tissue-specific manner. This will lead to a more comprehensive scientific basis for maximising the benefits of high-intensity exercise.
